# Hepatopulmonary Syndrome in a Thirteen Year Old Boy: A Case Report

**DOI:** 10.7759/cureus.5295

**Published:** 2019-08-01

**Authors:** Misbah Shahid, Asim Tameez Ud Din, Farooq Mohyud Din Chaudhary, Romaisa Malik, Ahsan Tameez-ud-din

**Affiliations:** 1 Gastroenterology, Nishtar Medical University & Hospital, Multan, PAK; 2 Internal Medicine, Rawalpindi Medical University, Rawalpindi, PAK

**Keywords:** hepatopulmonary syndrome, contrast echocardiography, cirrhosis

## Abstract

Hepatopulmonary syndrome (HPS) is a late complication associated with liver cirrhosis. Recent studies have suggested that it can also occur in non-cirrhotic portal hypertension. The criteria that need to be met for making the diagnosis of HPS include chronic liver disease, impaired gaseous exchange leading to hypoxemia and intrapulmonary vascular dilation. The pathophysiology of this disease includes mediators like nitric oxide (NO), and endothelial growth factors which play an important role in intrapulmonary dilation of vessels. This, in turn, leads to ventilation-perfusion mismatch which is the main etiology of pulmonary symptoms associated with this syndrome. The classical symptoms include dyspnea, orthodeoxia and platypnea. Contrast echocardiography has emerged to be a very sensitive test for its diagnosis. The timing of appearance of microbubbles help to differentiate between intracardiac and intrapulmonary shunting, with the latter being the hallmark of HPS. The only curative treatment available is liver transplantation. Here we present a case of a 13-year-old patient who presented in Nishtar hospital in Multan, Pakistan. He initially presented with signs of liver cirrhosis but no definite cause could be found. The patient didn’t come for a follow-up after that. Now he presented with signs of HPS and acute decompensated liver disease. HPS was confirmed on contrast echocardiography. This is a rare presentation of HPS in this age group.

## Introduction

Hepatopulmonary syndrome (HPS) is an unusual complication associated with liver cirrhosis. Its documented prevalance in literature is variable, ranging from 4-47% in cirrhotic patients [1]. Its diagnosis is formulated on the basis of clinical as well as examination findings. Classically, there should be evidence of underlying chronic liver disease, hypoxemia and dilation of intrapulmonary vessels in order to label the patient with HPS. The intrapulmonary shunting developed as a result of vessel dilation results in the ventilation-perfusion mismatch which manifests as pulmonary symptoms like dyspnea [2]. The management includes different pharmacological options including somatostatin analogs and steroids. However, these are found to be of minimal benefit. The only therapy which has shown significant improvement in these patients is liver transplantation [3]. Despite all the available interventions, HPS is considered a poor prognostic factor for patients with liver cirrhosis [4]. Here we present a case of a 13-year-old patient who presented in Nishtar hospital Multan, Pakistan with signs of HPS. This is a rare case of HPS in this age group.

## Case presentation

A 13-year-old boy presented in the emergency department at Nishtar hospital in 2017 with complaints of abdominal distension and upper gastrointestinal bleed (UGIB). Esophagogastroduodenoscopy (EGD) performed at that time showed bleeding at the esophageal varices. Band ligation was done to correct this. Workup at that time revealed the following investigations to be negative: hepatitis B surface antigen (HBsAg), antibodies to hepatitis C virus (anti-HCV), antinuclear factor (ANF), anti-mitochondrial antibodies (AMA), anti-smooth muscle antibody (ASMA), anti parietal cell antibodies and anti-liver kidney microsomal type 1 antibodies (anti-LKM1). Serum ceruloplasmin levels were 24 mg/dl (normal 20-40 mg/dl). The slit-lamp examination did not reveal Kayser Fleischer rings. Serum iron was 32 microgram/dl (normal, 65-175), total iron-binding capacity (TIBC) was 184 microgram/dl (normal, 250-400), transferrin saturation 17.4% (normal range 14-50%). Ultrasound (USG) of the abdomen showed coarse liver with splenomegaly and gross ascites. Ascitic fluid examination showed high serum ascites albumin gradient (SAAG) ratio. There was no evidence of spontaneous bacterial peritonitis (SBP). There was no history of alcohol intake, diabetes or any other co-morbid illness. After discharge, the patient did not get proper follow up for his liver disease and showed poor compliance to treatment.

In June 2019, he presented to the Nishtar hospital emergency department with complaints of worsening abdominal distension and exertional dyspnea for one month and black, tarry stools for two days. He became dyspneic even while going to the washroom. Attendants also noted that in the last few weeks the patient's hands, feet and parts of face would turn blue whenever he would perform mild to moderate exertion. The patient had no history of non-steroidal anti-inflammatory drugs (NSAIDs) intake. At the time of presentation, his blood pressure (BP) was 90/50 mmHg, pulse rate 102/min and a regular, respiratory rate of 30 breaths per minute at rest. The patient was pale and showed grade four clubbing on examination. His fingers, tip of nose and lips were cyanosed. Abdominal examination revealed splenomegaly and gross ascites. He was conscious and oriented. However, flapping tremors were present. His oxygen saturation (spO2) in the supine position was 85% which fell to 78% when the patient sat upright in bed (orthodeoxia). It was also observed that the patient's dyspnea and tachypnea worsened on sitting forward or standing due to which the patient preferred lying supine (platypnea). Patient's management in the emergency department included intravenous resuscitation with fluids, antibiotics, dextrose, terlipressin and oxygen therapy for his hypoxemia. Initial workup revealed: hemoglobin 7g/dl (normal 13-18 g/dl), total leukocyte count 12000 /mm^3^ (normal 4000-11000 /mm^3^), platelet count 100,000 /mm^3^ (normal 150,000-400,000 /mm^3^), serum bilirubin 0.3mg/dl (normal up to 1.2 mg/dl), aspartate aminotransferase (AST) 16 U/l (normal range, 10-40 U/l) and alanine aminotransferase (ALT) 39 U/l (normal range, 7-56 U/l), creatinine 0.8 mg/dl (normal 0.5-1.2 mg/dl), serum albumin 1.47 g/dl (normal 3.5-5.2 mg/dl), prothrombin time (PT) 15 seconds (control 12 sec), international normalized ratio (INR) 1.25. His child class was C (score 10). After cross-matching, packed red blood cells were transfused. After resuscitation, EGD was performed which showed grade two esophageal varices with stigmata of recent bleed. Endoscopic variceal band ligation was done. A diagnostic and therapeutic tap was performed. However, the patient did not show any improvement in dyspnea. Ascitic fluid showed high SAAG ratio. However, there was no evidence of SBP. USG of the abdomen showed coarse shrunken liver with splenomegaly and moderate to severe ascites. A doppler USG showed normal flow in the hepatic and portal veins.

Based on the history, examination and initial workup a provisional diagnosis of decompensated liver disease with ascites complicated by variceal bleeding and HPS was made. Further evaluation by transthoracic echocardiogram showed fair biventricular systolic function and mild pericardial effusion with an ejection fraction of 55% (normal 50-70%). For diagnostic confirmation, a bubble contrast Echocardiogram was performed which showed the appearance of microbubbles in the left atrium (LA) and left ventricle (LV) within three to five cardiac cycles after injection of agitated saline suggestive of intra-pulmonary shunts. Intra-cardiac shunts were absent on an echocardiography. A diagnosis of hepatopulmonary syndrome was made on the basis of the above-mentioned findings. The patient was put on continuous oxygen therapy as he was not able to maintain saturation without oxygen. A plan for liver biopsy was discussed with the patient and his family to find the cause of his liver disease. The patient as well his family were not willing to perform a liver biopsy at that time. The patient's condition improved gradually in a few days up on continuous oxygen therapy at 2-4 liters/min. The patient and his family were made aware of his condition and were referred to the national liver transplant center for live donor liver transplantation (LDLT). 

## Discussion

HPS is diagnosed when the triad of an underlying liver disease along with hypoxemia and evidence of intrapulmonary vessels dilation is present in a patient. This can also be associated with other classical features like platypnea and orthodeoxia which were seen in our patient [2]. The criterion was also fulfilled by our patient. Liver cirrhosis was evident by the findings of the ultrasound of the abdomen and endoscopy of the upper gastrointestinal tract. There was also hypoxemia (SpO_2_ of 78-84%) at the time of admission and echocardiogram showed findings suggestive of intrapulmonary shunting, making HPS a definitive diagnosis.

Although HPS is mainly associated with liver cirrhosis, there are multiple cases of patients with non-cirrhotic portal hypertension presenting with similar symptoms [5]. HPS can present as a late complication in young patients who are diagnosed with portal hypertension due to biliary atresia [6]. Our patient has cirrhotic portal hypertension but there was no associated co-morbid. The main pathophysiology associated with this syndrome is the intrapulmonary dilation of blood vessels. This is mainly accomplished by nitric oxide (NO). Other mediators like endothelial growth factor also play an important role by increasing vascular proliferation. These factors lead to a ventilation-perfusion mismatch which causes the pulmonary symptoms associated with it. The main diagnostic tests for HPS include contrast echocardiography, pulse oximetry, arterial blood gases (ABGs) and macro aggregated albumin lung perfusion scan (99mTc-MAA). Other less specific investigations include chest X-ray, chest CT-scan and routine baseline laboratory tests. Most of these investigations were done in our patient except 99mTc-MAA which was not available in our setting [7].

Contrast echocardiography is a very sensitive test for the diagnosis of HPS. It helps to differentiate between intrapulmonary and intracardiac shunting which aids in the diagnosis. In both types of patients, bubbles appear in the left side of heart but the difference is the time at which they are seen after injection of agitated saline. In cases of patients with an intracardiac shunt, they manifest within three heartbeats, which are in contrast to intrapulmonary shunting where appearance of bubbles is between four to six heartbeats. Our patient showed microbubbles in three to five cardiac cycles consolidating our diagnosis of HPS [8].

There are multiple case reports featuring the different clinical features and diagnostic tests of HPS [9,10]. In our case, the interesting aspect is the young age of the patient with no co-morbid.

The management of HPS includes both pharmacological and surgical options. Although there is the availability of different drugs like somatostatin analogs and immunosuppressive agents, neither has shown a significant mortality benefit. According to the European Association of Study of Liver (EASL), long term oxygen therapy is helpful in these patients. However, the only curative option is liver transplantation [3]. The major limitation of our case report is that we do not have good quality images of contrast echocardiography available to present in the article.

## Conclusions

Hepatopulmonary syndrome is a rare complication associated with cirrhotic as well as noncirrhotic portal hypertension. Cyanosis, platypnea, and orthodeoxia are the hallmark clinical features of HPS. The diagnostic criteria include underlying liver disease, hypoxemia and intrapulmonary dilation of vessels. Contrast echocardiography is the most sensitive test for its diagnosis. Without treatment, the prognosis is poor in these patients. No medical therapy has proven to be beneficial in this syndrome. EASL recommends long term oxygen therapy in these patients along with symptomatic treatment of other complications of cirrhosis. Definite therapy is liver transplantation. In this case report, we present a case of 13-year-old boy who presented with HPS. After initial management, he was referred to the national liver transplant center for liver transplantation.
